# Mercy Hospital Ophthalmic Clinic

**Published:** 1875-01-15

**Authors:** S. J. Jones


### MERCY HOSPITAL OPHTHALMIC CLINIC.

### SERVICE OF PROF. S. J. JONES, M.D.

*Reported by F. J. Huse, M.D.*

I.

T. R. H——; thirty-three; blacksmith's helper; healthy, and of temperate habits; while breaking off the end of a bar of iron, was forcibly struck in the left eye by a fragment weighing about two ounces. A jagged angle penetrated the cornea at its outer and lower margin, freely lacerating the iris and the capsule of the crystalline lens. Another sharp projection inflicted a wound in the sclerotica, through which a small portion of the pigmental layer of the choroid may be easily distinguished, owing to the gaping produced by the increased interocular tension.

Four days subsequently he makes his appearance, complaining of very severe supraorbital pain, great tenderness of the eye, and complete obstruction of vision, consequent upon traumatic cataract. The eyelids are ecchymosed, the conjunctiva is highly injected, and there is a bright red zone around the margin of the cornea. At the point of the corneal wound, there is an irregular conical bulging over a line in height and two lines in diameter, through the truncated apex of which there is hernia of the iris. The anterior chamber is very nearly filled with portions of the opaque lenticular substance, and the capsule is adherent to the upper and inner margin of the iris for a space nearly three lines in length.

There is ordered to be dropped into the eye, once daily, a solution of atropia (sulphate), five grains to the ounce of distilled water, for the purpose of dilating the pupil as widely as possible away from contact with the lens, and diminishing the ciliary disturbance. In addition, the patient is directed to use a collyrium containing two grains of zinc sulphate to the ounce of distilled water, and to make frequent applications to the lids, of thin pieces of muslin wet with cold water; at the same time, his bowels are to be kept freely open.

Oct. 14th.—There has been a subsidence of the pain, and under the influence of the atropia, the pupil has become irregularly dilated. Notwithstanding an improvement in the iritis, the capsule of the lens remains adherent to the posterior surface of the iris; the flakes of lenticular substance are being slowly absorbed, the hernia of the iris is lessened, but the eye is still inflamed and sensitive.

Oct. 21st.—An improvement has taken place in the general condition of the eye; and absorption of the opaque lenticular matter is occurring, especially in the outer portion of the pupil, so that the patient can now count fingers at the distance of three feet.

Dec. 1st.—Only a very small amount of lens matter remains in the anterior chamber, and the border of the iris has been gradually detached from the capsule of the lens, so that its outline is now perfectly natural, except at the point where hernia occurred. The hernial projection has been slowly diminished, until it is scarcely perceptible. A portion of the lens still occupies the posterior chamber, but vision is growing much clearer, and the process of absorption will continue until the removal of the entire lens shall have been effected; thus the injury which produces cataract, also affords the means of its removal. The admission of the aqueous humor, at first rendering the contents of the capsule opaque, promotes its final dissolution.

II.

S. R. M——; has severe pain, and considerable tinnitus in the right ear, from which she has been suffering since her exposure eight days ago, to a severe storm. Removing the numerous wrappings about her head which have been assisting the inflammatory process by keeping the parts hot and promoting decomposition of the discharge, the patient presents an example of those cases in which inflammation of the meatus is aggravated by the nature of the dressing. The entire auricle, which has been repeatedly smeared with goose oil and common salt, is much inflamed and excoriated. The meatus, which has been kept plugged with cotton saturated with the same compound, is narrowed concentrically throughout its entire extent; the tissues are œdematous and exceedingly tender; and the surface almost entirely stripped of its epidermis, looks red and roughened. After cleansing the meatus by syringing, the membrana tympani is found to be greatly thickened and hyperæmic. There is copious discharge. Upon catheterization, the eustachian tube is found to be moderately pervious. There is no inflammation of the fauces.

It is ordered that the parts be kept cleansed by repeated syringing with warm water; that the superfluous coverings be dispensed with; and that the meatus be filled twice daily with the following solution, slightly warmed:

℞.—Zinci sulph., grs. v.
Acidi carbol., (cryst.), grs. ii.
Glycerinæ, f ʒ ij.
Aquæ destill., f ℥ ij,—M.

Leeches are to be applied to the tragus and meatus, if the pain and inflammation continue.

III.

J. D. E——; twenty-seven; blacksmith; was struck in the left eye while engaged in driving a rivet by a small piece of iron, which pierced the cornea, passed through the pupil, and penetrated the crystalline lens. Severe inflammation and traumatic cataract have followed, and now five days after the injury was received, he presents himself for treatment.

Anæsthetizing the patient with chloroform, the lens is extracted by the modified operation of von Graefe; but careful search fails to reveal the presence within it of the foreign body. To obviate the risk of sympathetic inflammation of the other eye, enucleation is performed; and in the posterior portion of the vitreous humor, is found the particle of iron.

